# Holocene centennial variability in sea surface temperature and linkage with solar irradiance

**DOI:** 10.1038/s41598-022-19050-6

**Published:** 2022-09-03

**Authors:** Si Woong Bae, Kyung Eun Lee, Tae Wook Ko, Ryoung Ah Kim, Young-Gyu Park

**Affiliations:** 1 Ocean Science and Technology School, Korea Maritime and Ocean University, Busan, 49112 South Korea; 2 Department of Ocean Science, Korea Maritime and Ocean University, Busan, 49112 South Korea; 3 Ocean Circulation Research Center, Korea Institute of Ocean Science and Technology, Busan, 49111 South Korea

**Keywords:** Palaeoceanography, Palaeoclimate

## Abstract

The climate periodically fluctuates on various time scales, however, there remains a lack of consensus on the centennial-scale variabilities and associated driving force. A continuous high-resolution sea surface temperature (SST) record allows for the detection of centennial-scale fluctuations. This study presents a high-resolution SST record covering the last 10,000 years based on the analysis of the alkenone unsaturation index in marine sediment cores off the southwest coast of the Korean Peninsula. Alkenone SST's spectral and wavelet analysis revealed significant periodicities of 414, 190, 135, 102, and 89 years at a > 90% confidence level. These cycles exhibit extreme proximity to the solar activity cycles of 353, 206 (Suess/de Vries cycles), 130, and 104–87 years (Gleissberg cycles), suggesting that the multidecadal to centennial variations in SST are linked to solar forcing. To the best of our knowledge, this is the first high-resolution Holocene SST record that all solar activity cycles on centennial scale match, suggesting centennial-scale variability in the climate system and illustrating the role of solar activity on SST change in the mid-latitude region of the Northern Hemisphere.

## Introduction

The climate periodically fluctuates on various time scales such as the glacial-interglacial cycles associated with orbital forcing^1–3^, millennial variations such as the Dansgaard–Oeschger cycle associated with ocean thermohaline circulation^4,5^, and decadal and interannual variations such as the Pacific Decadal Oscillation^6^, North Atlantic Oscillation^7^, Atlantic Multi-decadal Variability^8^, and the El Niño-Southern Oscillation^9^. However, there remains a lack of consensus on the centennial-scale variabilities in climate: centennial variations and major forcing controlling their periodic fluctuations remain debated^10,11^. The instrumental observation data are only available for the past 100 years. In part, the temporal resolution of paleo-proxy records is not high enough to resolve the centennial-scale variations.

It is evident that solar activity has varied on decadal to millennial time scales throughout the Holocene^12^. A solar cycle (i.e., the Schwabe cycle) lasts 11 years, which can be observed by the number of sunspots^13^. The polarity of the magnetic fields of the sunspot changes every two Schwabe cycles. This 22-year magnetic cycle is known as the Hale cycle^14^. The amplitude modulation of the Schwabe cycles throughout 60–120 years is known as the Gleissberg cycle^15^. In addition, longer solar cycles have been reported: the 210-year Suess/de Vries cycle^16^ associated with the recurrence of grand minima, the 1000-year Eddy cycle^17^, and the 2,400-year Hallstatt cycle^18^. The Holocene total solar irradiance (TSI) data inferred from the production rates of radiocarbon in tree ring and ^10^Be obtained from ice cores^19^ indicate that the periodicities of 361, 206, 149, 130, 105, and 88 years were significant, which suggests the presence of centennial variability in solar activity during the Holocene.

Recent research has focused on reconstructing high-resolution climate variations in East Asia during the Holocene based on various proxies (e.g., cave stalagmite δ^18^O, pollen, planktonic foraminiferal δ^18^O)^20–23^. These studies suggest that climate changes in this region did not monotonically change during the Holocene, rather periodically fluctuated on a centennial scale. Additionally, the authors indicated that the periodic climate changes might have been associated with solar cycles^20–23^. However, spectral analyses of climate proxy records for the Holocene have shown that their variables share only one or two solar activity cycles^20–23^. The periodicity of the matching cycles also differs in each study. High-resolution (5 years) stalagmite δ^18^O data from Chinese Dongge Cave^20^ indicate changes in δ^18^O values of precipitation related to variations in East Asian summer monsoon intensity. The record shares the cycles of 206 and 159 years with TSI, suggesting that the solar changes were partly responsible for the centennial-scale changes in summer monsoon^20^. Planktonic foraminiferal (*Neogloboquadrina incompta*) δ^18^O data (resolution: ~ 30 years) from the northeastern part of Japan^21^, which reflect changes in East Asian winter monsoon intensity, shares the cycles of 750 and 470 years. High-resolution (~ 20 years) pollen records from a Chinese lake (Xiaolongwan)^22^ indicate changes in the vertical distribution of vegetation and air temperature; the records share a cycle of 500 years. High-resolution (~ 24 years) tree pollen index of warmness from a Korean swamp (Mulyoungari)^23^ also reflects the changes in air temperature and shares a cycle of 145 years.

High-resolution Holocene sea surface temperature (SST) records in East Asia are scarce. Due to challenges such as scarcity, resolution, continuity, and uncertainty of SST proxy records, it was difficult to identify the multi-decadal- and centennial-scale variations in SST over the Holocene from proxy records. Even today, a scarcity of high-resolution SST records over the Holocene impedes further progress toward identifying the natural variability in SST on multi-decadal and centennial scales and understanding the relations with climate forcing. To clarify the centennial-scale SST variations, it is necessary to reconstruct high-resolution SST records from quantitative temperature proxy such as alkenone. In this study, we reconstruct a high-resolution (i.e., approximately 10–20-year) SST composite covering the Holocene using the alkenone unsaturation index obtained from the marine sediments of two deep-drilled cores recovered from the Heuksan mud belt (HMB) located off the southwest coast of the Korean Peninsula. The centennial-scale SST variability was identified from the alkenone records. Importantly, linkage to changes in solar activity was investigated. Examining their relationship can elucidate the influence of centennial-scale changes in solar activity on climate changes in mid-latitude East Asia.

## Results

**Figure 1:**
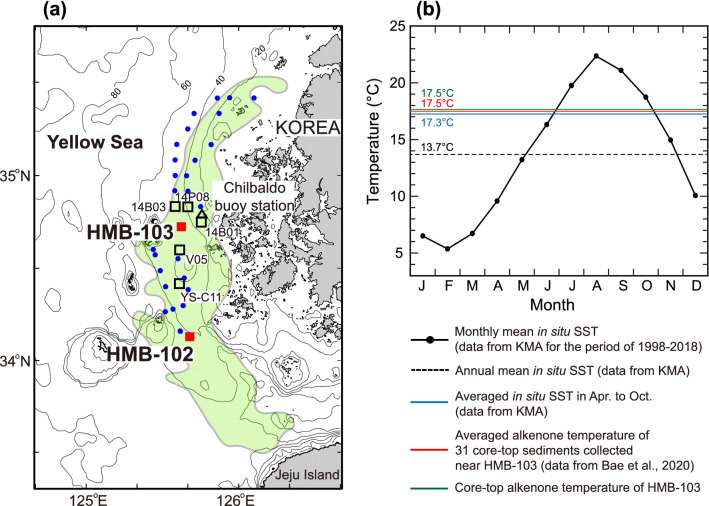
Study area and modern monthly mean sea surface temperatures. (**a**) Bathymetric map of the study area (contours in m) and core locations. Map was generated using MATLAB R2017a with ETOPO1 data (https://www.ngdc.noaa.gov/mgg/global/). The Heuksan Mud Belt (HMB) is indicated with green shading. Red squares indicate the location of the two deep-drilled cores (HMB-102, HMB-103). Black squares indicate the location of the box cores (14HMB-B01, 14HMB-B03), piston cores (14HMB-P08, YS-C11^33^), and vibro core (HMB V-05) used for ^210^Pb analysis. Blue dots indicate the location of core-top sediments used for alkenone analysis. Black triangle indicates the Chilbaldo Buoy Station of the Korea Meteorological Administration. (**b**) Monthly mean SSTs at the Chilbaldo Buoy Station (data from KMA database, observed from 1998 to 2018). Black dashed line indicates the annual averaged in situ SST. Blue thick line indicates the averaged in situ SST from April to October. Thick green and red lines indicate the alkenone temperature of HMB-103 core-top and the averaged alkenone temperature of 31 core-top sediments collected near HMB-103, respectively.

The HMB runs in a north–south direction along the south-western coast of Korea (Fig. 1a). Water depths in the HMB range from 20 m in the north to 110 m in the south. This study used marine sediment from the deep-drilled cores HMB-103 (34°43.50'N, 125°37.51'E; water depth of 48 m) and HMB-102 (34°7.96’N, 125°40.94’E, water depth: 57 m). The HMB is characterised by high sedimentation rates (approximately 0.2 cm/yr), allowing for the reconstruction of the SST record at a high resolution. To investigate changes of monthly mean SST at the study area, in situ SST data from Chilbaldo buoy station (dataset from the Korea Meteorological Administration, KMA) for the period 1998–2018 was examined (Fig. 1b)^24^. The lowest SST (5.4 °C) occurs in February, whereas the highest (22.4 °C) occurs in August. The annual mean SST is 13.7 °C.

SST at the study area is strongly influenced by the seasonality of two atmospheric circulation systems: the mid-latitude Westerly Jet (WJ) and the East Asian monsoon. During winter, the main route of the WJ lies in the south of the Himalayas (< 30°N)^25,26^, and the dry and cold terrestrial air mass anchored in Siberia expands southward^27^. Conversely, during summer, the WJ moves to the north of the Tibetan Plateau (> 42°N). The wet and warm subtropical air mass from the Northwest Pacific overarches the East Asian marginal sea areas. Change in atmospheric heat flux associated with seasonality of the two-circulation system is the primary cause of SST variations in this shallow area^28^. The extended winter regime with both dry and cold air and stronger wind speed accompanied by the southward shift of the WJ can maintain the vertical contrast between the local ocean and the atmosphere and cause excessive heat loss to the atmosphere, thereby decreased the SST^27,29,30^. In the meantime, the persisting summer regime associated with the northward migration of Westerly Jet would reduce the heat loss to the atmosphere, thereby increases the SST.

**Figure 2:**
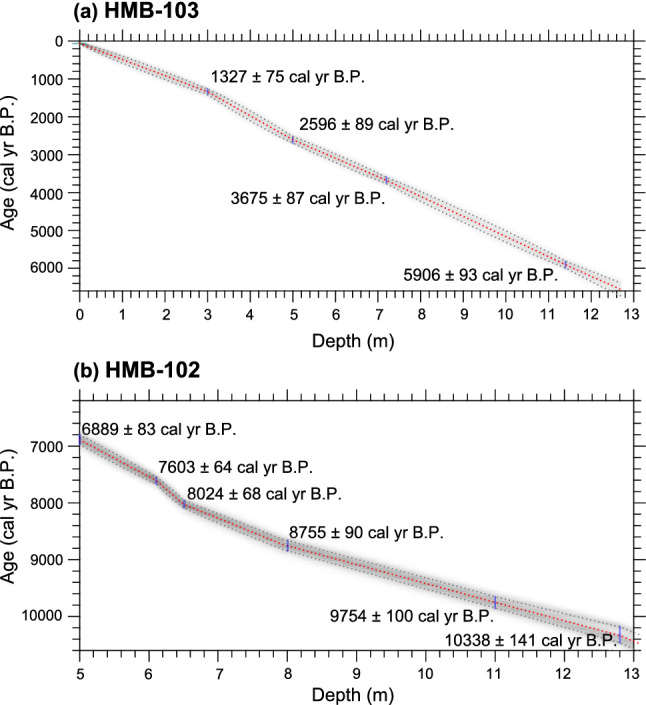
Age-depth model. Bayesian age-depth models of the cores (**a**) HMB-103 and (**b**) HMB-102 established using Bacon 2.5 software^32^. Dashed red and grey lines indicate the best model based on the weighted mean age for each depth and its 1σ confidence intervals, respectively.

Radiocarbon dating of ten samples from cores HMB-102 (six samples) and -103 (four samples) has been previously measured using benthic foraminifera and bivalve shell in Holocene muddy sediments^31^. The age-depth model for each core was constructed using the Bacon 2.5 package in R^32^ with the Marine20 radiocarbon calibration curve and the ΔR value of  284 ± 39 years (Fig. 2). The Bacon method simulates the accumulation rates of core sediments based on Bayesian statistics and calculates the ages and uncertainties (1σ) of sediment deposits. Core HMB-103 covers the last 6.5 cal kyr B.P.; while, core HMB-102 covers the period between 6.9 and 10 cal kyr B.P.. The results of the ^210^Pb dating of the HMB-103 and other core-top sediments collected near the HMB-103 were examined. The ^210^Pb and ^226^Ra activities were measured at the Korea Basic Science Institute (KBSI). The observed excess ^210^Pb activities in the box cores indicate that the core-top surface sediments near the HMB-103 are modern-day sediments, which is in line with previous findings^33^. However, the ^210^Pb dating of HMB-103 indicates that the upper most sediment appears to be at least 111 years older than the sampling year of 2013 (see “Method”). According to the age model, the core HMB-103 and -102 have a high sedimentation rate (approximately 0.2 cm/yr), which allows for the SST reconstruction at a high resolution (approximately 10–20 years) for the Holocene.

A new high-resolution alkenone SST composite covering the Holocene was constructed by combining records from cores HMB-103 and -102. For the SST reconstruction of HMB-103, newly analysed alkenone data (269 samples) were added to the previously published temperature data (80 samples)^24^, hence a total of 349 samples were used in this study. For the HMB-102, 188 new samples were added to the previous 41 samples^24^. Since we collected sediment samples at 3 cm interval, there could be bioturbation effects. In this case, the temperature signal would be smoothed and/or reduced by mixing of the sediments. The error range of the alkenone temperature assessment for replicate samples of the two kinds of homogeneous laboratory-standard marine sediments exhibited less than ± 0.05 °C (n = 90) and ± 0.07 °C (n = 7) at 95% confidence level, respectively. The alkenone SST estimates obtained from the 31 core-top sediments collected near HMB-103 were compared with in situ temperatures^24^. The results indicated that the alkenone unsaturation index effectively represented the averaged SST from April to October in this area (Fig. 1b).

**Figure 3:**
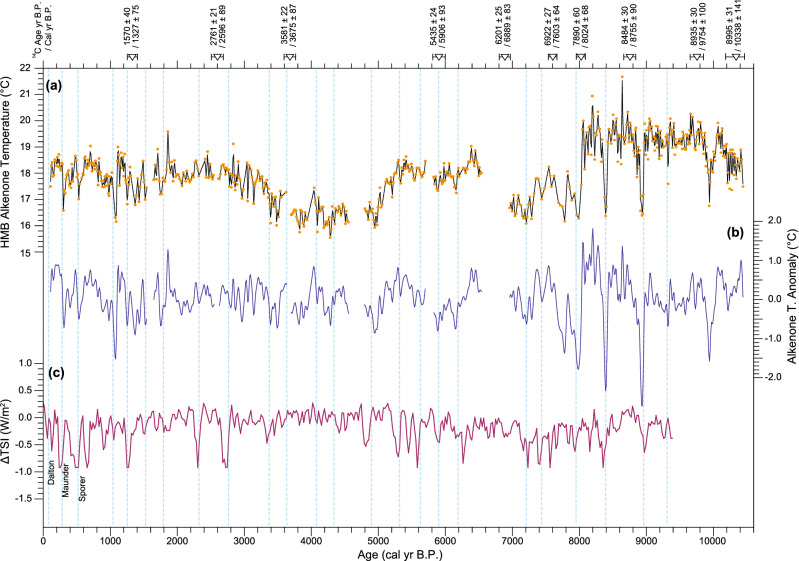
Time series of alkenone sea surface temperature and solar activity. (**a**) High-resolution alkenone-based SSTs versus calendar age for the entire Holocene. Orange dots indicate the original SST records reconstructed from HMB-102 and -103. The black line indicates the 10-year interpolated alkenone SST for statistical analyses. (**b**) The timeseries of 50–2000-year band-pass filtered alkenone SSTs. (**c**) Total solar irradiance (TSI) anomalies reconstructed from principal component analysis of ^14^C in tree ring and ^10^Be in ice cores^19^. Anomaly values are relative to the value of 1986 (1365.57 W/m^2^). Inverted triangles indicate radiocarbon dating^31^. Vertical dashed lines (cyan) show possible correlations between low SSTs and weak solar activity.

**Figure 4:**
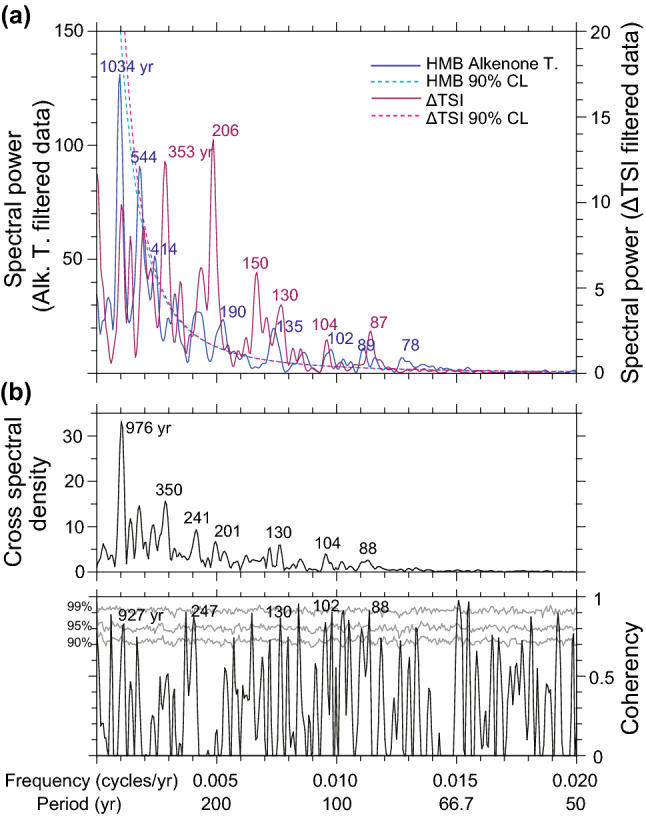
Spectrum power and coherency between alkenone temperature and solar activity. Spectral analysis result for (**a**) 50–2000-year band-pass filtered alkenone SST (Blue) and ΔTSI (purple) with a 90% confidence level (dashed line). Calculations were conducted using the REDFIT 38 software^48^. (**b**) The cross-spectral coherency between band-pass filtered data with 90, 95, and 99% confidence level (grey line), conducted using the REDFIT-X software^49^.

**Figure 5:**
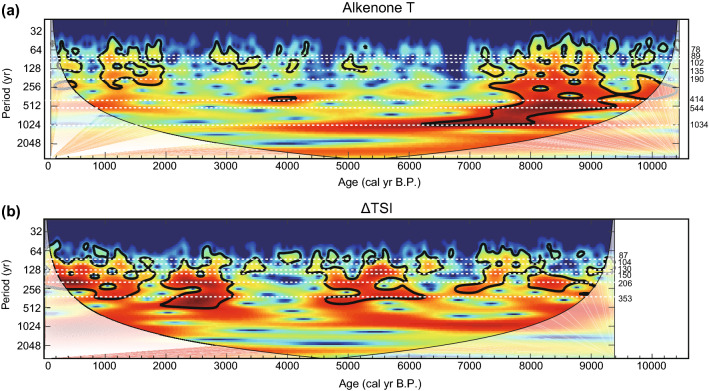
Wavelet analysis of alkenone sea surface temperature and solar activity. Continuous Morlet transforms wavelet spectra for 50–2000 years band-pass filtered (**a**) alkenone SST and (**b**) ΔTSI. White dashed line indicates significant periodicities. Black contour indicates a 95% confidence level.

Figure 3a,b shows the resulting high-resolution SST record, reconstructed from alkenone analysis of the HMB-102 and -103 cores. The 10-year interpolated alkenone SSTs are listed in Supplementary Table S1. The essential observed characteristic was the periodic SST fluctuations on a multidecadal- to millennial-scale. Although the amplitude of variations often range within ± 1 °C (Fig. 3a), these periodic fluctuations were distinct, considering the error range of SST reconstructions from homogeneous marine sediment (< ± 0.1 °C). SSTs were relatively warm during the early Holocene (8–10 cal kyr B.P.), then cooled during the period of 6.5–8 cal kyr B.P., then warm again between 5 and 6.5 cal kyr B.P. (Fig. 3a). There were several distinct and rapid cooling events with a large amplitude up to 3 °C during the period of 7–9 cal kyr B.P. (Fig. 3a). In a previous study on the reconstruction of low-resolution (~ 50 years) alkenone SSTs from cores HMB-102 and -103, Bae et al.^24^ demonstrated that the cold period dropped by 2 °C for the period of 3–5 cal kyr B.P. was a regional pattern in the mid-latitudes of East Asia via comparison with other regional temperature records. Two cooling periods (3–5 cal kyr B.P. and ~ 7–8 cal kyr B.P.) were possibly associated with millennial variations. To remove the cooling patterns on a millennial scale and investigate centennial variations, we extracted 50–2000 years periodicities from the raw dataset by applying a band-pass filter (Fig. 3b). The spectral analysis of band-pass filtered SSTs revealed significant (> 90% confidence level) periodicities of 544, 414, 190, 135, 102, and 89 years (Fig. 4a). In addition, 1034-year cycle variability seems to exist (Fig. 4a). We also conducted wavelet analysis on the Holocene alkenone SSTs (Fig. 5a). The cycles of 544, 414, 190, 135, 102, 89, and 78 years were significant for the interval of 7–9 cal kyr B.P. (Fig. 5a). Another characteristic of the wavelet analysis results is that 1034-year cycles predominated during the early to mid- Holocene (Fig. 5a). The periodic centennial-scale fluctuations of alkenone SSTs with the periodicities of 190, 135, 102, and 89 years have been strengthened after 2 cal kyr B.P. (Fig. 5a). Overall, the composite SST record indicated the occurrence of centennial-scale variability over the last 10,000 years.

We used the Holocene total solar irradiance (TSI) data obtained by Steinhilber et al.^19^ (Fig. 3c) for comparison with our composite SST record. TSI data have been reconstructed based on the principal component analysis of production rates of ^14^C in tree ring and ^10^Be obtained from ice cores collected in Greenland and Antarctica. Variations in TSI during the Holocene exhibit periodic fluctuations on multidecadal to millennial scales, although the magnitudes were less than 1 W/m^2^ (Fig. 3c). The spectral analysis on band-pass filtered (50–2000 years) TSI data revealed that the periodicities of 353, 206, 150, 130, 104, and 87 years were significant (> 90% confidence level) (Fig. 4a). The wavelet analysis of the TSI data demonstrated that the periodic centennial-scale fluctuations of solar activity have been strengthened for the periods of 0–3, 4.5–6, and 7–9 cal kyr B.P. (Fig. 5b).

The composite SST record was compared to the variations in solar activity over the Holocene (Fig. 3), showing a good agreement at the centennial scale. Periods of low SST corresponded approximately to those of low TSI within the estimated age error (± 60–90 years). In particular, the periods of minimum solar activity known as Dalton (~ 1820 CE), Maunder (~ 1680 CE), and Spörer (~ 1470 CE), which have been previously recognised from sunspot observation data covering the last 500 years, correspond to cold intervals in our SST composite. We compared the cross spectrum analysis results obtained from these two datasets (Fig. 4b). Cycles of 89 and 102 years in the SST record could have been associated with those of 87 and 104 years in the TSI record (i.e., Gleissberg cycles). Additionally, the 190-year cycles observed in HMB-103 were similar to the 206-year TSI cycles (i.e., Suess/de Vries cycles). Finally, the 414-year cycle may reflect 353-year TSI cycles (unnamed cycles). The results of the coherence calculation between the SSTs and the TSI confirm the correspondence of the periodicities between them (Fig. 4b). The periodicities of 88, 102, 130, 247, and 927 years are highly coherent between SST and TSI (> 99% Monte Carlo false alarm level) (Fig. 4b). The simultaneous occurrence of cold SSTs and low TSI, combined with their similar cyclicity, indicates that the solar activity may have influenced the centennial-scale SST variability in the study area.

## Discussion

In this study, alkenone palaeothermometry was used to reconstruct high-resolution SST records. Our SSTs indicate that centennial variations were evident throughout the Holocene, and were readily comparable to the solar activity. However, changes in solar activity were small (within 1 W/m^2^) during the Holocene. It remains uncertain how such a small change might have affected the Holocene climate changes^34,35^. Previous studies have explained that the small change in solar activity is amplified via climatic processes (e.g. stratospheric response, and ocean–atmosphere circulation responses) and plays an important role on changes in global climate^34,36^. Intense solar activity and increased ultraviolet solar radiation (UV) stimulate ozone production and UV absorption in the stratosphere, causing the stratosphere to warm differentially concerning latitude. This causes atmospheric circulation changes in the stratosphere, propagated downward to the troposphere^36^. The Hadley circulation expands when the solar activity is intense and shrinks when it is weak^37,38^. Finally, this might be associated with the northward or southward migration of the WJ^39^.

Seasonal SSTs at the study area are influenced by (i) the north–south migration of WJ location and monsoon-related wind system and (ii) associated increase or loss of sensible and latent heat flux at the surface. Even during the same season, the variations in SST in the study area may be related to the latitude where the WJ is placed. In previous studies, the 200 hPa wind vector corresponding to the WJ was reanalysed from the wind field data provided by NCEP and ERA-interim and compared with observed SST near the study area^30,40^. In a year when the WJ locates in relatively low latitudes from autumn to winter, the SSTs in the East Sea, Yellow Sea, and the East China Sea tend to decrease overall compared to other years. In contrast, in a year when the WJ moves to high latitudes, the SSTs tend to increase. The same mechanism can be applied to the centennial variations in TSI and SST during the Holocene. The small changes in solar activity on a centennial-scale during the Holocene might have been amplified by the WJ over East Asia, causing centennial-scale SST changes in the study area. Intense solar activity associated with the northward migration of the WJ would be related to warm SSTs.

**Figure 6:**
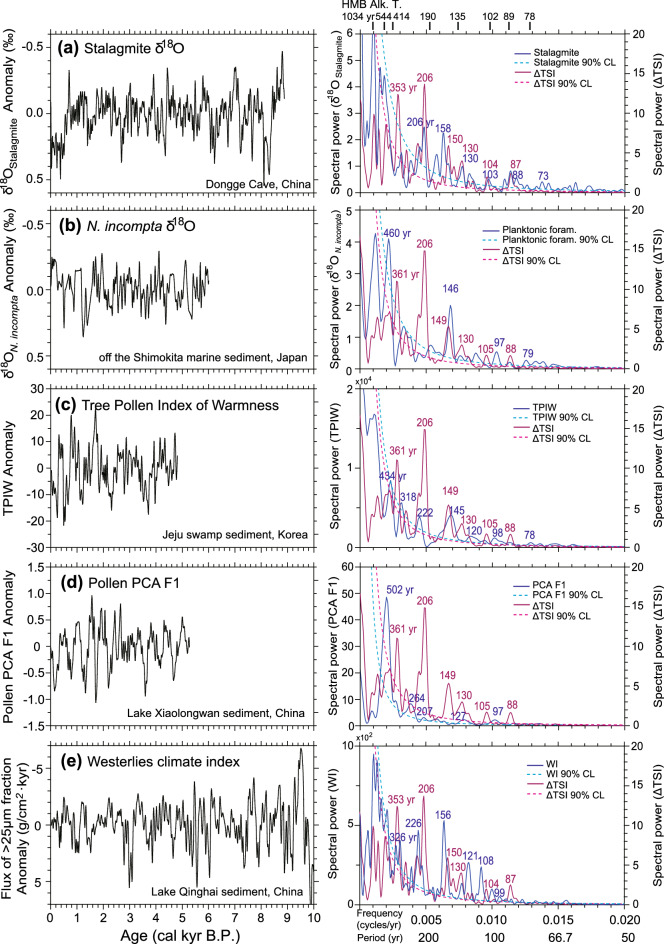
Time series and spectrum power of centennial-scale climate variabilities. (**a**) Stalagmite δ^18^O records from Dongge Cave, China^20^. (**b**) δ^18^O of planktonic foraminifera (*Neogloboquadrina incompta*) in Core SK-2 collected off the Shimokita Peninsula, Japan^21^. (**c**) Calculated tree pollen index of warmness (TPIW) at the Mulyoungari swamp in Jeju Island, Korea^23^. (**d**) First principal component (PCA F1) of terrestrial pollen percentage records at Lake Xiaolongwan, China^22^. (**e**) Flux of > 25 μm fraction anomaly at Lake Qinghai, China^41^. All proxy records were 50–2000 years band-pass filtered. Blue line indicates proxy spectral power; purple line indicates ΔTSI spectral power; black vertical bar on the top indicates significant periodicities of the HMB alkenone SSTs.

High-resolution climate variations were inferred from several other terrestrial proxy records in East Asia^20–23,41^. We conducted spectral analysis of previously published proxy records filtered in a same way of this study, and compared the results with those of TSI. The spectral analysis of the high-resolution (5 years) stalagmite δ^18^O data from a Chinese Dongge cave^20^, recording the variations in the amount of precipitation and thus East Asian summer monsoon intensity, indicates the periodicities of 206, 158, 130, 103, 88, and 73 years (Fig. 6a). These periodicities exhibit proximity to the TSI cycles of 210 (Suess/de Vries cycles), 150, 130, and 120–60 years (Gleissberg cycles). Notably, our alkenone SST records represent the averaged SSTs from April to October in the study area, which is close to summer average (Fig. 1b). Comparisons of our SST records to Dongge cave records suggested that the centennial-scale variations in SST reconstructed in our study were similar to variations in the East Asian summer monsoon intensity and TSI for all cycles of the Holocene. The result of the spectral analysis of the high-resolution (~ 24 years) pollen records from Korean swamp^23^, which indicate warmness in air temperature, showed the significant periodicities of 222, 145, 120, and 98 years, which is in turn consistent with our SST centennial cycles (Fig. 6c). In particular, a high-resolution reconstruction of the speculated WJ changes during the Holocene, inferred from grain-size analysis of Lake Qinghai (located in the north-eastern Tibetan Plateau) sediments^41^, also indicates that the WJ tended to shift northward when the solar activity was intense. The Gleissberg cycle of the solar activity is observable in the spectrum analysis of the Holocene WJ record^41^ (Fig. 6e). Meanwhile, planktonic foraminiferal (*Neogloboquadrina incompta*) δ^18^O data (resolution: ~ 30 years) from the northeast part of Japan^21^, which reflect changes in East Asian winter monsoon intensity, share cycles of 460, 146, 97, and 79 years (Fig. 6b). High-resolution (~ 20 years) pollen records from a Chinese lake (Xiaolongwan)^22^, which indicate changes in the vertical distribution of vegetation and air temperature, share cycles of 502 and 264 years (Fig. 6d).

The millennial-scale variations in the alkenone SSTs might be related to the millennial-scale variations in the location of the main route of the WJ. The main route of WJ during the early Holocene (8–10 cal kyr B.P.) is considered to have lain at further high latitude (40–50°N) than it does now^42^. In contrast, the main route of WJ was located at a lower latitude (< 30°N) between 3–5 cal kyr B.P. than its present-day location^24,43^. During the late Holocene (0–3 cal kyr B.P.), it has lied on the present latitude (30–42°N) where the study area is located^42^. Alkenone SST records indicate the occurrence of warm conditions during the early Holocene, cold ones during the period of 6–8 cal kyr B.P., and warming again during the middle Holocene (5–6 cal kyr B.P). During the period of 3–5 cal kyr B.P., it was cold, and warm again thereafter. These support the relationship between the migration of the WJ and SST changes.

In summary, we produced high-resolution (i.e., approximately ten years) composite SST records covering the last 10,000 years in the mid-latitude region of the Northern Hemisphere. The SST records exhibit an overall in-phase relationship between the SST and TSI changes at the centennial scale. Periods of low SST correspond approximately to those of low TSI within the estimated age error (± 60–90 years). Solar cycles, such as those of 353, 206 (Suess/de Vries cycles), 130, and 104–87 years (Gleissberg cycles), are evident in the SST records. This implies the linkage of the centennial SST variability to solar forcing. The WJ might have amplified the small changes in centennial-scale solar activity during the Holocene over East Asia. The north–south migration of WJ location and changes in monsoon-related wind system leads to increase or loss of sensible and latent heat flux at the surface, causing centennial-scale SST changes in the study area. Here, our high-resolution SST data fluctuate on a centennial scale, illustrating the critical role of TSI variability on periodic Holocene climate changes in the mid-latitudes.

## Methods

The cores HMB-102 and -103 were collected in 2013 by the Korean Institute of Geoscience and Mineral Resources (KIGAM), using the Chinese drillship KAN 407. We used Holocene muddy sediments collected from the interval of 0–13 m of the HMB-103 and 5–13 m for the HMB-102 for the alkenone analyses. A previous study presented low resolution of the alkenone-derived SST records from the same cores^24^. For this study, additional samples were analysed from cores HMB-102 and -103. For Core HMB-102, 188 new samples were added to the previous 41 samples, hence a total of 229 samples were used in this study. Conversely, for HMB-103, 269 new samples were added to the previous 80 samples, hence a total of 349 samples were used. The sediment samples were collected at 3–4 cm intervals in the form of 1 cm thick slices.

For SST reconstruction, we conducted C_37_ alkenone analysis using freeze-dried sediments (2 g). The C_37_ alkenones were measured at the Korea Maritime and Ocean University. The sediments were extracted using an accelerated solvent extractor (ASE-200, Dionex Corporation) with the solvent (CH_2_Cl_2_:MeOH, 99:1) maintained at 100 ℃ and 1500 psi for 10 min. The extracted organic matter was cleaned by elution with CH_2_Cl_2_ (500 μL × 3) through a silica cartridge (Silica SPE cartridge, 100 mg/mL, Agilent Technologies). Subsequently, 0.1 M potassium hydroxide (KOH) was added to the samples and maintained at 70 ℃ for 2 h to allow for saponification. The alkenone fraction was obtained by partitioning into hexane. After being concentrated under a gentle N_2_ flux, the alkenones were separated from the final extract using a gas chromatograph (Agilent GC 7890B) equipped with a flame ionization detector and fused silica capillary column (J & W DB-1, 0.32 mm × 0.25 μm × 60 m, Agilent Technologies). The degree of unsaturation ($U_{{{37}}}^{{{\text{K}}^{^{\prime}} }}$) was calculated using Eq. (1)^44^, and the alkenone temperature was reconstructed using Eq. (2)^45^.

1 $$U_{{{37}}}^{{{\text{K}}^{^{\prime}} }} = \, \left[ {{\text{C}}_{{{37}:{2}}} } \right]/\left( {\left[ {{\text{C}}_{{{37}:{2}}} } \right] + \left[ {{\text{C}}_{{{37}:{3}}} } \right]} \right)$$

2 $${\text{T }} = \left( {U_{{{37}}}^{{{\text{K}}^{^{\prime}} }} - 0.0{39}} \right)/0.0{34}$$

The age of the two deep-drilled cores HMB-102 and -103 was determined via radiocarbon dating as previously reported^31^. Accelerator mass spectrometry (AMS) measured the ^14^C ages of the benthic foraminifera and bivalve shells in the cores at the Institute of Geological Nuclear Science in New Zealand and the KIGAM in Korea, respectively. The AMS-measured ^14^C ages of cores HMB-102 and -103 were converted into calendar ages using Bacon 2.5 software in R^32^ with the Marine20 calibration curve^46^. The used marine reservoir correction (ΔR) is  284 ± 39 years, which represents the mean value of four ΔR values published in the Yellow Sea^47^ (http://calib.org/marine/). We used the section of 0–13 m of core HMB-103, covering the last 6.5 cal kyr B.P. with the uncertainty of 60–90 years (Fig. 2a). For the HMB-102, we used the section from 5 to 13 m, corresponding the time-interval of 6.9–10.3 cal kyr B.P. with an uncertainty of 64–141 years (Fig. 2b). By combining the records from the two cores, one time series of the high-resolution SST data for the entire Holocene were reconstructed. The results of ^210^Pb dating from the core HMB-103 and the core-top sediments of 2 box cores (14HMB-B01, 14HMB-B03), piston (14HMB-P08) and vibro (HMB V-05) cores obtained from the vicinity of the HMB-103 site (Fig. 1a) are presented in Supplementary Table S2. The total ^210^Pb and ^226^Ra activities were estimated from the Korea Basic Science Institute (KBSI). The excess ^210^Pb values of the coretop sediments of the box cores were 3.5–4.2 dpm/g, similar to the results of a previous study^33^. These values indicate that the coretop sediments of the box cores are modern-day sediments. However, the ^210^Pb dating of the piston and vibro cores show that the total ^210^Pb and ^226^Ra activities of the coretop sediments were similar, suggesting that the sediments were not modern. The ^210^Pb dating of the HMB-103 indicates that the total ^210^Pb and ^226^Ra activities of the upper part sediments (~ 30 cm deep) were also similar, suggesting that the sediments were older than the sampling year of 2013. They appear to be at least 111 years (an age equivalent to 5 times the half-life of ^210^Pb, 22.2 years) older than the sampling year of 2013. Hence, we assume that the coretop sediments correspond to the year 1902. The calendar age of HMB-103 is 1.3 cal kyr B.P. at 3 m and 5.9 cal kyr B.P. at 11.4 m with an uncertainty of 75 and 93 years, respectively (Fig. 2a).

The data were further analysed to investigate centennial variations using band-pass filtering (50–2000 years). Spectral analyses of the records were conducted using the REDFIT 38 software^48^ with the following parameter settings: Monte Carlo simulations = 1000, oversampling factor = 4, highest frequency factor = 1, Welch-overlapped-segment-averaging segment (n50) = 3, and Welch window = 1. Cross spectral analysis was carried out using the REDFIT-X software^49^ on the same terms. Additionally, continuous Morlet transform wavelet analyses were performed in MATLAB R2017a using the toolbox of Grinsted et al.^50^.

## Supplementary Information

Supplementary Information.

## Data Availability

Data relevant to this study are available in the Supplementary Information and will be archived at the National Oceanic and Atmospheric Administration National Centres for Environmental Information (NCEI).
