# Supplementary material for: Holocene centennial variability in sea surface temperature and linkage with solar irradiance
Source: Sci Rep. 2022 Sep 3;12:15046. doi: 10.1038/s41598-022-19050-6 (PMC9440922; doi:10.1038/s41598-022-19050-6)
Supplement: Supplementary file 1 — Supplementary Information. [file 41598_2022_19050_MOESM1_ESM.docx]

Supplementary Information for

**“Holocene centennial variability in sea surface temperature and linkage with solar irradiance”**

**Supplementary Table S1.** Alkenone-based SST records of cores HMB-102 and -103

| Age  (yr B.P.) | Alk. T.  (℃) | Age  (yr B.P.) | Alk. T.  (℃) | Age  (yr B.P.) | Alk. T.  (℃) | Age  (yr B.P.) | Alk. T.  (℃) |
| --- | --- | --- | --- | --- | --- | --- | --- |
| 110 | 17.7 | 500 | 18.1 | 890 | 17.6 | 1280 | 17.6 |
| 120 | 18.3 | 510 | 17.9 | 900 | 17.6 | 1290 | 18.1 |
| 130 | 18.4 | 520 | 17.7 | 910 | 17.5 | 1300 | 18.1 |
| 140 | 18.1 | 530 | 17.1 | 920 | 17.8 | 1310 | 17.8 |
| 150 | 17.9 | 540 | 17.5 | 930 | 17.6 | 1320 | 17.7 |
| 160 | 18.0 | 550 | 17.6 | 940 | 17.7 | 1330 | 17.5 |
| 170 | 18.5 | 560 | 17.7 | 950 | 17.9 | 1340 | 17.3 |
| 180 | 18.5 | 570 | 17.8 | 960 | 17.9 | 1350 | 17.1 |
| 190 | 18.4 | 580 | 17.9 | 970 | 17.4 | 1360 | 17.0 |
| 200 | 18.4 | 590 | 18.0 | 980 | 17.1 | 1370 | 16.8 |
| 210 | 18.6 | 600 | 18.0 | 990 | 17.5 | 1380 | 17.0 |
| 220 | 18.3 | 610 | 18.4 | 1000 | 17.8 | 1390 | 17.2 |
| 230 | 18.5 | 620 | 18.6 | 1010 | 17.6 | 1400 | 17.5 |
| 240 | 18.5 | 630 | 18.5 | 1020 | 17.9 | 1410 | 17.7 |
| 250 | 18.2 | 640 | 18.2 | 1030 | 17.8 | 1420 | 17.3 |
| 260 | 18.1 | 650 | 18.2 | 1040 | 17.5 | 1430 | 17.0 |
| 270 | 18.3 | 660 | 18.4 | 1050 | 17.2 | 1440 | 17.3 |
| 280 | 18.4 | 670 | 18.4 | 1060 | 16.8 | 1450 | 17.6 |
| 290 | 17.9 | 680 | 18.3 | 1070 | 16.5 | 1460 | 17.8 |
| 300 | 16.6 | 690 | 18.4 | 1080 | 16.3 | 1470 | 18.0 |
| 310 | 17.2 | 700 | 18.8 | 1090 | 16.4 | 1480 | 18.2 |
| 320 | 17.3 | 710 | 18.7 | 1100 | 17.8 | 1490 | 18.5 |
| 330 | 17.4 | 720 | 18.3 | 1110 | 18.8 | 1500 | 17.8 |
| 340 | 17.8 | 730 | 18.1 | 1120 | 18.9 | 1510 | 17.4 |
| 350 | 17.9 | 740 | 18.3 | 1130 | 18.3 | 1520 | 17.0 |
| 360 | 18.0 | 750 | 18.3 | 1140 | 17.6 | 1530 | 17.2 |
| 370 | 18.0 | 760 | 18.2 | 1150 | 18.2 | 1540 | 17.4 |
| 380 | 18.0 | 770 | 18.2 | 1160 | 18.6 | 1650 | 17.8 |
| 390 | 17.6 | 780 | 18.5 | 1170 | 18.6 | 1660 | 18.1 |
| 400 | 17.6 | 790 | 18.4 | 1180 | 18.3 | 1670 | 18.3 |
| 410 | 17.6 | 800 | 18.2 | 1190 | 18.6 | 1680 | 18.0 |
| 420 | 17.8 | 810 | 17.9 | 1200 | 18.1 | 1690 | 17.9 |
| 430 | 18.2 | 820 | 17.6 | 1210 | 18.3 | 1700 | 18.5 |
| 440 | 17.7 | 830 | 18.4 | 1220 | 18.4 | 1710 | 18.8 |
| 450 | 17.9 | 840 | 18.2 | 1230 | 17.7 | 1720 | 18.3 |
| 460 | 18.2 | 850 | 18.0 | 1240 | 17.5 | 1730 | 17.8 |
| 470 | 18.4 | 860 | 18.0 | 1250 | 17.0 | 1740 | 17.4 |
| 480 | 18.6 | 870 | 17.7 | 1260 | 17.2 | 1750 | 17.2 |
| 490 | 18.5 | 880 | 17.5 | 1270 | 17.7 | 1760 | 17.2 |

**Supplementary Table S1.** Alkenone-based SST records of cores HMB-102 and -103 (Continued)

| Age  (yr B.P.) | Alk. T.  (℃) | Age  (yr B.P.) | Alk. T.  (℃) | Age  (yr B.P.) | Alk. T.  (℃) | Age  (yr B.P.) | Alk. T.  (℃) |
| --- | --- | --- | --- | --- | --- | --- | --- |
| 1770 | 17.4 | 2140 | 18.1 | 2510 | 17.9 | 2960 | 17.2 |
| 1780 | 17.7 | 2150 | 18.0 | 2520 | 17.8 | 2970 | 17.1 |
| 1790 | 17.8 | 2160 | 17.8 | 2530 | 17.8 | 2980 | 17.3 |
| 1800 | 17.8 | 2170 | 17.8 | 2540 | 17.9 | 2990 | 17.5 |
| 1810 | 17.7 | 2180 | 17.7 | 2630 | 17.8 | 3000 | 17.7 |
| 1820 | 17.8 | 2190 | 17.7 | 2640 | 17.9 | 3010 | 17.9 |
| 1830 | 17.9 | 2200 | 17.7 | 2650 | 18.1 | 3020 | 18.0 |
| 1840 | 18.1 | 2210 | 17.8 | 2660 | 18.3 | 3030 | 18.2 |
| 1850 | 18.8 | 2220 | 17.9 | 2670 | 18.3 | 3040 | 18.4 |
| 1860 | 19.6 | 2230 | 17.9 | 2680 | 18.3 | 3050 | 17.1 |
| 1870 | 19.0 | 2240 | 17.9 | 2690 | 18.3 | 3060 | 17.5 |
| 1880 | 18.4 | 2250 | 18.1 | 2700 | 18.2 | 3070 | 18.2 |
| 1890 | 18.2 | 2260 | 18.4 | 2710 | 18.1 | 3080 | 18.2 |
| 1900 | 18.1 | 2270 | 18.4 | 2720 | 18.0 | 3090 | 18.2 |
| 1910 | 18.3 | 2280 | 18.5 | 2730 | 18.2 | 3100 | 18.1 |
| 1920 | 18.3 | 2290 | 18.3 | 2740 | 18.2 | 3110 | 18.0 |
| 1930 | 17.9 | 2300 | 18.2 | 2750 | 18.1 | 3120 | 17.8 |
| 1940 | 17.8 | 2310 | 18.0 | 2760 | 18.1 | 3130 | 17.6 |
| 1950 | 18.2 | 2320 | 17.9 | 2770 | 18.0 | 3140 | 16.8 |
| 1960 | 18.4 | 2330 | 18.0 | 2780 | 17.6 | 3150 | 17.2 |
| 1970 | 18.1 | 2340 | 18.0 | 2790 | 17.9 | 3160 | 17.6 |
| 1980 | 17.9 | 2350 | 18.1 | 2800 | 17.7 | 3170 | 17.7 |
| 1990 | 17.9 | 2360 | 18.2 | 2810 | 17.5 | 3180 | 17.7 |
| 2000 | 17.9 | 2370 | 18.2 | 2820 | 17.9 | 3190 | 17.8 |
| 2010 | 18.0 | 2380 | 18.3 | 2830 | 18.6 | 3200 | 18.0 |
| 2020 | 17.9 | 2390 | 18.4 | 2840 | 18.7 | 3210 | 18.0 |
| 2030 | 17.8 | 2400 | 18.4 | 2850 | 17.7 | 3220 | 17.9 |
| 2040 | 17.8 | 2410 | 18.5 | 2860 | 17.5 | 3230 | 17.8 |
| 2050 | 17.7 | 2420 | 18.3 | 2870 | 17.5 | 3240 | 17.8 |
| 2060 | 17.6 | 2430 | 18.0 | 2880 | 17.9 | 3250 | 17.8 |
| 2070 | 17.5 | 2440 | 18.2 | 2890 | 17.7 | 3260 | 17.9 |
| 2080 | 17.8 | 2450 | 18.7 | 2900 | 17.9 | 3270 | 17.7 |
| 2090 | 18.0 | 2460 | 18.4 | 2910 | 18.4 | 3280 | 17.4 |
| 2100 | 17.9 | 2470 | 17.9 | 2920 | 18.0 | 3290 | 17.2 |
| 2110 | 17.9 | 2480 | 18.1 | 2930 | 17.8 | 3300 | 17.5 |
| 2120 | 17.8 | 2490 | 18.3 | 2940 | 17.7 | 3310 | 17.6 |
| 2130 | 17.8 | 2500 | 18.1 | 2950 | 17.5 | 3320 | 17.5 |

**Supplementary Table S1.** Alkenone-based SST records of cores HMB-102 and -103 (Continued)

| Age  (yr B.P.) | Alk. T.  (℃) | Age  (yr B.P.) | Alk. T.  (℃) | Age  (yr B.P.) | Alk. T.  (℃) | Age  (yr B.P.) | Alk. T.  (℃) |
| --- | --- | --- | --- | --- | --- | --- | --- |
| 3330 | 17.7 | 3770 | 16.6 | 4140 | 16.6 | 4510 | 16.1 |
| 3340 | 17.6 | 3780 | 16.4 | 4150 | 16.7 | 4520 | 16.4 |
| 3350 | 17.2 | 3790 | 16.3 | 4160 | 16.7 | 4530 | 16.4 |
| 3360 | 17.2 | 3800 | 16.2 | 4170 | 16.7 | 4540 | 16.2 |
| 3370 | 17.2 | 3810 | 16.1 | 4180 | 16.5 | 4550 | 16.2 |
| 3380 | 16.8 | 3820 | 15.9 | 4190 | 15.9 | 4560 | 16.1 |
| 3390 | 16.3 | 3830 | 15.8 | 4200 | 16.0 | 4570 | 16.2 |
| 3400 | 16.5 | 3840 | 16.6 | 4210 | 16.1 | 4800 | 16.6 |
| 3410 | 17.2 | 3850 | 16.6 | 4220 | 16.1 | 4810 | 16.6 |
| 3420 | 17.0 | 3860 | 16.5 | 4230 | 16.1 | 4820 | 16.5 |
| 3430 | 16.8 | 3870 | 16.3 | 4240 | 16.0 | 4830 | 16.5 |
| 3440 | 16.8 | 3880 | 16.1 | 4250 | 16.2 | 4840 | 16.5 |
| 3450 | 17.0 | 3890 | 16.0 | 4260 | 16.3 | 4850 | 16.3 |
| 3460 | 17.1 | 3900 | 16.2 | 4270 | 16.2 | 4860 | 16.5 |
| 3470 | 16.9 | 3910 | 16.5 | 4280 | 15.7 | 4870 | 16.8 |
| 3480 | 16.2 | 3920 | 16.3 | 4290 | 15.7 | 4880 | 16.7 |
| 3490 | 16.3 | 3930 | 16.2 | 4300 | 15.9 | 4890 | 16.6 |
| 3500 | 16.5 | 3940 | 16.2 | 4310 | 16.5 | 4900 | 16.5 |
| 3510 | 16.3 | 3950 | 16.4 | 4320 | 16.6 | 4910 | 16.6 |
| 3520 | 16.6 | 3960 | 16.5 | 4330 | 16.3 | 4920 | 16.5 |
| 3530 | 17.0 | 3970 | 16.6 | 4340 | 16.2 | 4930 | 16.3 |
| 3540 | 17.3 | 3980 | 16.7 | 4350 | 16.1 | 4940 | 16.1 |
| 3550 | 17.1 | 3990 | 16.8 | 4360 | 16.5 | 4950 | 16.0 |
| 3560 | 17.1 | 4000 | 16.9 | 4370 | 16.6 | 4960 | 16.3 |
| 3570 | 17.1 | 4010 | 17.0 | 4380 | 16.6 | 4970 | 16.3 |
| 3580 | 17.2 | 4020 | 17.2 | 4390 | 16.5 | 4980 | 16.1 |
| 3590 | 17.2 | 4030 | 17.3 | 4400 | 16.6 | 4990 | 16.3 |
| 3600 | 17.2 | 4040 | 17.4 | 4410 | 16.7 | 5000 | 16.8 |
| 3610 | 17.2 | 4050 | 17.3 | 4420 | 16.7 | 5010 | 17.0 |
| 3620 | 17.3 | 4060 | 16.9 | 4430 | 16.6 | 5020 | 17.2 |
| 3630 | 17.2 | 4070 | 16.7 | 4440 | 16.4 | 5030 | 17.3 |
| 3710 | 16.4 | 4080 | 16.6 | 4450 | 16.5 | 5040 | 16.8 |
| 3720 | 16.5 | 4090 | 16.0 | 4460 | 16.6 | 5050 | 16.7 |
| 3730 | 16.5 | 4100 | 16.3 | 4470 | 16.8 | 5060 | 16.8 |
| 3740 | 16.6 | 4110 | 17.0 | 4480 | 16.8 | 5070 | 17.0 |
| 3750 | 16.6 | 4120 | 16.7 | 4490 | 16.7 | 5080 | 17.1 |
| 3760 | 16.6 | 4130 | 16.5 | 4500 | 16.4 | 5090 | 17.3 |

**Supplementary Table S1.** Alkenone-based SST records of cores HMB-102 and -103 (Continued)

| Age  (yr B.P.) | Alk. T.  (℃) | Age  (yr B.P.) | Alk. T.  (℃) | Age  (yr B.P.) | Alk. T.  (℃) | Age  (yr B.P.) | Alk. T.  (℃) |
| --- | --- | --- | --- | --- | --- | --- | --- |
| 5100 | 17.5 | 5470 | 18.1 | 5960 | 17.6 | 6330 | 18.2 |
| 5110 | 17.6 | 5480 | 18.1 | 5970 | 17.7 | 6340 | 18.4 |
| 5120 | 17.7 | 5490 | 18.3 | 5980 | 18.2 | 6350 | 18.4 |
| 5130 | 17.8 | 5500 | 18.3 | 5990 | 18.1 | 6360 | 18.4 |
| 5140 | 17.7 | 5510 | 18.2 | 6000 | 17.8 | 6370 | 18.5 |
| 5150 | 17.6 | 5520 | 18.0 | 6010 | 17.9 | 6380 | 18.8 |
| 5160 | 17.6 | 5530 | 17.8 | 6020 | 18.0 | 6390 | 19.0 |
| 5170 | 17.6 | 5540 | 17.7 | 6030 | 18.1 | 6400 | 18.7 |
| 5180 | 17.7 | 5550 | 17.8 | 6040 | 18.0 | 6410 | 18.6 |
| 5190 | 17.8 | 5560 | 18.0 | 6050 | 18.1 | 6420 | 18.5 |
| 5200 | 17.5 | 5570 | 18.1 | 6060 | 18.3 | 6430 | 18.3 |
| 5210 | 17.2 | 5580 | 18.2 | 6070 | 18.1 | 6440 | 18.5 |
| 5220 | 17.5 | 5590 | 18.2 | 6080 | 17.9 | 6450 | 18.8 |
| 5230 | 17.8 | 5600 | 18.2 | 6090 | 17.9 | 6460 | 18.6 |
| 5240 | 18.0 | 5610 | 18.2 | 6100 | 17.8 | 6470 | 18.4 |
| 5250 | 18.0 | 5620 | 18.1 | 6110 | 17.9 | 6480 | 18.4 |
| 5260 | 18.0 | 5630 | 18.0 | 6120 | 17.8 | 6490 | 18.1 |
| 5270 | 18.0 | 5640 | 17.9 | 6130 | 17.6 | 6500 | 17.9 |
| 5280 | 18.2 | 5650 | 17.9 | 6140 | 17.5 | 6510 | 18.2 |
| 5290 | 18.4 | 5660 | 17.9 | 6150 | 17.5 | 6520 | 18.1 |
| 5300 | 18.5 | 5670 | 18.0 | 6160 | 17.3 | 6530 | 18.0 |
| 5310 | 18.2 | 5680 | 18.0 | 6170 | 17.5 | 6540 | 18.0 |
| 5320 | 17.8 | 5690 | 18.1 | 6180 | 17.9 | 6960 | 16.7 |
| 5330 | 17.7 | 5700 | 18.4 | 6190 | 18.1 | 6970 | 16.8 |
| 5340 | 17.8 | 5710 | 18.4 | 6200 | 18.1 | 6980 | 16.8 |
| 5350 | 18.1 | 5720 | 18.4 | 6210 | 17.9 | 6990 | 16.7 |
| 5360 | 18.1 | 5850 | 17.7 | 6220 | 17.9 | 7000 | 16.6 |
| 5370 | 18.0 | 5860 | 17.8 | 6230 | 18.0 | 7010 | 16.9 |
| 5380 | 18.4 | 5870 | 17.5 | 6240 | 18.1 | 7020 | 17.1 |
| 5390 | 18.4 | 5880 | 17.4 | 6250 | 18.1 | 7030 | 16.7 |
| 5400 | 18.2 | 5890 | 17.3 | 6260 | 18.2 | 7040 | 16.4 |
| 5410 | 18.5 | 5900 | 17.5 | 6270 | 18.2 | 7050 | 16.4 |
| 5420 | 18.5 | 5910 | 17.7 | 6280 | 18.2 | 7060 | 16.5 |
| 5430 | 18.2 | 5920 | 17.9 | 6290 | 18.1 | 7070 | 16.8 |
| 5440 | 18.3 | 5930 | 17.9 | 6300 | 18.0 | 7080 | 17.0 |
| 5450 | 18.3 | 5940 | 17.7 | 6310 | 18.1 | 7090 | 17.1 |
| 5460 | 18.1 | 5950 | 17.6 | 6320 | 18.2 | 7100 | 17.0 |

**Supplementary Table S1.** Alkenone-based SST records of cores HMB-102 and -103 (Continued)

| Age  (yr B.P.) | Alk. T.  (℃) | Age  (yr B.P.) | Alk. T.  (℃) | Age  (yr B.P.) | Alk. T.  (℃) | Age  (yr B.P.) | Alk. T.  (℃) |
| --- | --- | --- | --- | --- | --- | --- | --- |
| 7110 | 16.8 | 7480 | 17.4 | 7850 | 17.6 | 8220 | 19.2 |
| 7120 | 16.8 | 7490 | 17.5 | 7860 | 17.4 | 8230 | 18.7 |
| 7130 | 16.8 | 7500 | 17.7 | 7870 | 17.2 | 8240 | 19.3 |
| 7140 | 16.6 | 7510 | 17.8 | 7880 | 17.1 | 8250 | 19.5 |
| 7150 | 16.4 | 7520 | 17.9 | 7890 | 17.0 | 8260 | 19.5 |
| 7160 | 16.3 | 7530 | 18.1 | 7900 | 17.0 | 8270 | 20.0 |
| 7170 | 16.3 | 7540 | 18.2 | 7910 | 17.1 | 8280 | 20.2 |
| 7180 | 16.5 | 7550 | 18.2 | 7920 | 17.2 | 8290 | 19.8 |
| 7190 | 16.6 | 7560 | 18.0 | 7930 | 17.2 | 8300 | 19.7 |
| 7200 | 16.3 | 7570 | 17.8 | 7940 | 16.9 | 8310 | 19.5 |
| 7210 | 16.1 | 7580 | 17.6 | 7950 | 16.7 | 8320 | 18.9 |
| 7220 | 16.3 | 7590 | 17.5 | 7960 | 16.4 | 8330 | 18.6 |
| 7230 | 16.6 | 7600 | 17.7 | 7970 | 16.4 | 8340 | 19.2 |
| 7240 | 16.8 | 7610 | 17.8 | 7980 | 16.3 | 8350 | 18.8 |
| 7250 | 16.9 | 7620 | 17.9 | 7990 | 16.3 | 8360 | 18.2 |
| 7260 | 17.1 | 7630 | 17.8 | 8000 | 16.5 | 8370 | 17.5 |
| 7270 | 16.8 | 7640 | 17.6 | 8010 | 16.9 | 8380 | 17.0 |
| 7280 | 16.5 | 7650 | 17.5 | 8020 | 17.2 | 8390 | 16.5 |
| 7290 | 16.3 | 7660 | 17.3 | 8030 | 17.7 | 8400 | 16.6 |
| 7300 | 16.6 | 7670 | 17.2 | 8040 | 19.1 | 8410 | 17.2 |
| 7310 | 16.9 | 7680 | 17.0 | 8050 | 19.8 | 8420 | 18.4 |
| 7320 | 17.1 | 7690 | 16.9 | 8060 | 20.0 | 8430 | 19.3 |
| 7330 | 17.3 | 7700 | 16.8 | 8070 | 19.1 | 8440 | 19.6 |
| 7340 | 17.5 | 7710 | 16.8 | 8080 | 18.2 | 8450 | 19.5 |
| 7350 | 17.8 | 7720 | 16.8 | 8090 | 18.9 | 8460 | 19.4 |
| 7360 | 17.8 | 7730 | 16.8 | 8100 | 19.4 | 8470 | 19.2 |
| 7370 | 17.8 | 7740 | 16.7 | 8110 | 19.5 | 8480 | 19.3 |
| 7380 | 17.8 | 7750 | 16.7 | 8120 | 19.1 | 8490 | 19.6 |
| 7390 | 17.8 | 7760 | 16.6 | 8130 | 18.8 | 8500 | 20.0 |
| 7400 | 17.6 | 7770 | 16.4 | 8140 | 19.5 | 8510 | 20.0 |
| 7410 | 17.5 | 7780 | 16.2 | 8150 | 19.8 | 8520 | 19.5 |
| 7420 | 17.3 | 7790 | 16.5 | 8160 | 19.7 | 8530 | 19.5 |
| 7430 | 17.1 | 7800 | 17.0 | 8170 | 19.2 | 8540 | 19.9 |
| 7440 | 17.0 | 7810 | 17.4 | 8180 | 19.0 | 8550 | 20.0 |
| 7450 | 17.2 | 7820 | 17.9 | 8190 | 20.5 | 8560 | 19.6 |
| 7460 | 17.3 | 7830 | 18.0 | 8200 | 20.6 | 8570 | 18.8 |
| 7470 | 17.3 | 7840 | 17.8 | 8210 | 20.0 | 8580 | 18.8 |

**Supplementary Table S1.** Alkenone-based SST records of cores HMB-102 and -103 (Continued)

| Age  (yr B.P.) | Alk. T.  (℃) | Age  (yr B.P.) | Alk. T.  (℃) | Age  (yr B.P.) | Alk. T.  (℃) | Age  (yr B.P.) | Alk. T.  (℃) |
| --- | --- | --- | --- | --- | --- | --- | --- |
| 8590 | 19.5 | 8960 | 18.2 | 9330 | 18.4 | 9700 | 19.8 |
| 8600 | 19.4 | 8970 | 19.5 | 9340 | 19.5 | 9710 | 19.4 |
| 8610 | 19.1 | 8980 | 19.5 | 9350 | 19.9 | 9720 | 19.1 |
| 8620 | 18.6 | 8990 | 18.8 | 9360 | 20.0 | 9730 | 18.9 |
| 8630 | 19.7 | 9000 | 19.5 | 9370 | 19.5 | 9740 | 19.1 |
| 8640 | 21.5 | 9010 | 19.4 | 9380 | 19.4 | 9750 | 19.4 |
| 8650 | 20.0 | 9020 | 18.8 | 9390 | 19.0 | 9760 | 19.5 |
| 8660 | 19.3 | 9030 | 19.1 | 9400 | 19.6 | 9770 | 19.7 |
| 8670 | 19.2 | 9040 | 19.9 | 9410 | 19.9 | 9780 | 19.7 |
| 8680 | 19.2 | 9050 | 20.0 | 9420 | 19.1 | 9790 | 19.2 |
| 8690 | 19.4 | 9060 | 19.9 | 9430 | 19.1 | 9800 | 19.6 |
| 8700 | 19.7 | 9070 | 19.7 | 9440 | 19.1 | 9810 | 19.9 |
| 8710 | 20.0 | 9080 | 19.8 | 9450 | 19.1 | 9820 | 19.6 |
| 8720 | 20.3 | 9090 | 19.3 | 9460 | 19.2 | 9830 | 19.4 |
| 8730 | 19.8 | 9100 | 19.2 | 9470 | 19.1 | 9840 | 19.2 |
| 8740 | 19.3 | 9110 | 19.4 | 9480 | 19.2 | 9850 | 18.9 |
| 8750 | 19.6 | 9120 | 19.1 | 9490 | 19.3 | 9860 | 19.1 |
| 8760 | 19.9 | 9130 | 19.1 | 9500 | 19.2 | 9870 | 19.5 |
| 8770 | 19.7 | 9140 | 19.6 | 9510 | 19.1 | 9880 | 18.9 |
| 8780 | 19.2 | 9150 | 19.5 | 9520 | 19.3 | 9890 | 18.5 |
| 8790 | 19.2 | 9160 | 19.1 | 9530 | 19.1 | 9900 | 18.8 |
| 8800 | 19.4 | 9170 | 19.7 | 9540 | 19.3 | 9910 | 18.3 |
| 8810 | 19.0 | 9180 | 19.4 | 9550 | 19.5 | 9920 | 18.0 |
| 8820 | 18.7 | 9190 | 18.6 | 9560 | 19.3 | 9930 | 18.0 |
| 8830 | 19.1 | 9200 | 19.3 | 9570 | 18.9 | 9940 | 16.9 |
| 8840 | 19.7 | 9210 | 19.6 | 9580 | 18.9 | 9950 | 17.6 |
| 8850 | 19.1 | 9220 | 19.0 | 9590 | 18.9 | 9960 | 18.4 |
| 8860 | 17.9 | 9230 | 19.0 | 9600 | 19.4 | 9970 | 18.5 |
| 8870 | 18.8 | 9240 | 19.2 | 9610 | 19.4 | 9980 | 18.2 |
| 8880 | 18.9 | 9250 | 19.0 | 9620 | 19.2 | 9990 | 18.0 |
| 8890 | 18.3 | 9260 | 19.0 | 9630 | 19.2 | 10000 | 18.6 |
| 8900 | 18.7 | 9270 | 19.0 | 9640 | 19.5 | 10010 | 18.7 |
| 8910 | 17.0 | 9280 | 19.0 | 9650 | 19.1 | 10020 | 18.6 |
| 8920 | 16.9 | 9290 | 19.1 | 9660 | 20.3 | 10030 | 18.7 |
| 8930 | 16.8 | 9300 | 19.3 | 9670 | 19.4 | 10040 | 19.0 |
| 8940 | 16.4 | 9310 | 19.5 | 9680 | 19.8 | 10050 | 19.4 |
| 8950 | 16.8 | 9320 | 18.3 | 9690 | 20.1 | 10060 | 19.6 |

**Supplementary Table S1.** Alkenone-based SST records of cores HMB-102 and -103 (Continued)

| Age  (yr B.P.) | Alk. T.  (℃) | Age  (yr B.P.) | Alk. T.  (℃) | Age  (yr B.P.) | Alk. T.  (℃) | Age  (yr B.P.) | Alk. T.  (℃) |
| --- | --- | --- | --- | --- | --- | --- | --- |
| 10070 | 19.8 | 10170 | 18.9 | 10270 | 18.0 | 10370 | 18.4 |
| 10080 | 19.1 | 10180 | 18.7 | 10280 | 18.0 | 10380 | 18.3 |
| 10090 | 19.5 | 10190 | 19.0 | 10290 | 18.8 | 10390 | 18.1 |
| 10100 | 19.5 | 10200 | 18.8 | 10300 | 18.4 | 10400 | 18.5 |
| 10110 | 18.9 | 10210 | 17.6 | 10310 | 18.1 | 10410 | 18.8 |
| 10120 | 19.4 | 10220 | 18.0 | 10320 | 18.7 | 10420 | 18.6 |
| 10130 | 19.7 | 10230 | 18.8 | 10330 | 18.2 | 10430 | 18.1 |
| 10140 | 19.1 | 10240 | 17.7 | 10340 | 17.9 | 10440 | 17.6 |
| 10150 | 18.7 | 10250 | 18.4 | 10350 | 18.1 |  |  |
| 10160 | 18.9 | 10260 | 18.8 | 10360 | 18.5 |  |  |

**Supplementary Table S2.** ^210^Pb dating from the core HMB-103 and other cores.

| Core ID | Core Type | Sample Depth  (cm) | Total ^210^Pb (dpm/g) | ^226^Ra (dpm/g) | Excess ^210^Pb (dpm/g) |
| --- | --- | --- | --- | --- | --- |
|  |  |  |  |  |  |
| 14HMB-B01 | Box core | 0-1 | 5.8 | 1.6 | 4.2 |
| 14HMB-B03 | Box core | 0-1 | 4.7 | 1.2 | 3.5 |
| 14HMB-P08 | Piston core | 0-1 | 1.8 | 1.1 | 0.7 |
| HMB V-05 | Vibro core | 0-1 | 1.3 | 1.1 | 0.1 |
| HMB-103 | Deep-drilled core | 17-18 | 1.9 | 1.3 | 0.6 |
| HMB-103 | Deep-drilled core | 20-27 | 1.7 | 1.4 | 0.3 |
| HMB-103 | Deep-drilled core | 29-30 | 1.9 | 1.3 | 0.6 |
| HMB-103 | Deep-drilled core | 32-33 | 2.0 | 1.5 | 0.4 |
|  |  |  |  |  |  |
